# Advancements in Microfluidic Cassette-Based iMiDEV™ Technology for Production of L-[^11^C]Methionine and [^11^C]Choline

**DOI:** 10.3390/ph17020250

**Published:** 2024-02-15

**Authors:** Hemantha Mallapura, Laurent Tanguy, Samin Mahfuz, Lovisa Bylund, Bengt Långström, Christer Halldin, Sangram Nag

**Affiliations:** 1 Department of Clinical Neuroscience, Center for Psychiatry Research, Karolinska Institutet and Stockholm County Council, SE-17176 Stockholm, Sweden; hemantha.mallapura@ki.se (H.M.); mahfuz@kth.se (S.M.); christer.halldin@ki.se (C.H.); 2 Business Unit Nuclear Medicine, PMB-Alcen, Route des Michels CD56, F-13790 Peynier, France; ltanguy@pmb-alcen.com; 3 Department of Radiopharmacy, Karolinska University Hospital, SE-17176 Stockholm, Sweden; lovisa.bylund@regionstockholm.se; 4 Department of Medicinal Chemistry, Uppsala University, SE-75123 Uppsala, Sweden; bengt.langstrom@kemi.uu.se

**Keywords:** positron emission tomography, iMiDEV, microfluidic cassette, batch-type microfluidics, radiosynthesizer, radiosynthesis, L-[^11^C]methionine, [^11^C]choline, radiotracers

## Abstract

Microfluidic technology is a highly efficient technique used in positron emission tomography (PET) radiochemical synthesis. This approach enables the precise control of reactant flows and reaction conditions, leading to improved yields and reduced synthesis time. The synthesis of two radiotracers, L-[^11^C]methionine and [^11^C]choline, was performed, using a microfluidic cassette and an iMiDEV^TM^ module by employing a dose-on-demand approach for the synthesis process. We focused on optimizing the precursor amounts and radiosynthesis on the microfluidic cassette. L-[^11^C]methionine and [^11^C]choline were synthesized using a microreactor filled with a suitable resin for the radiochemical reaction. Trapping of the [^11^C]methyl iodide, its reaction, and solid-phase extraction purification were performed on a microreactor, achieving radiochemical yields of >80% for L-[^11^C]methionine and >60% for [^11^C]choline (*n* = 3). The total synthesis time for both the radiotracers was approximately 20 min. All quality control tests complied with the European Pharmacopeia standards. The dose-on-demand model allows for real-time adaptation to patient schedules, making it suitable for preclinical and clinical settings. Precursor optimization enhanced the cost efficiency without compromising the yield. The importance of dose-on-demand synthesis and optimized precursor utilization to produce L-[^11^C]methionine and [^11^C]choline was emphasized in this study. The results demonstrated the feasibility of dose-on-demand adaptations for clinical applications with reduced precursor quantities and high radiochemical yields.

## 1. Introduction

Positron emission tomography (PET) is a powerful tool that offers insights into the molecular and physiological processes underlying various diseases [1]. It can provide diagnostic information about the homeostatic system without altering its function [2]. Additionally, various types of information can be collected using PET, including the staging of diseases, the correct selection of pharmacological or radionuclide therapies, responses to treatments and radiotherapy planning, and correct theragnostic approaches [3,4]. Additionally, it has been utilized as a tool for development in drug discovery, even in different disciplines, such as research and clinical trials [5].

Currently, PET radiotracers are produced in centralized, advanced nuclear medicine laboratories and then distributed to nearby clinics. However, many clinics have limited access to radiotracers to meet the immediate needs of their patients, and some specialized tracers may not be accessible due to the short half-life of radionuclides (Carbon-11; 20.34 min) [6,7]. The need for advanced, automated, and efficient radiotracer production has driven the development of innovative microfluidic techniques, offering promising solutions to overcome the existing challenges and fill the gap [8]. Microfluidic techniques offer significant advantages by enabling real-time and patient-specific radiotracer production such as the dose-on-demand approach [9]. Optimizing precursor quantities and establishing automated microfluidic synthesis protocols using the microfluidic cassette are crucial steps toward realizing the full potential of these batch-type microfluidic techniques to assess their suitability for dose-on-demand production.

^11^C-based radiopharmaceuticals are valuable for clinical applications because of their ability to provide additional diagnostic information about oncological and neurodegenerative disorders [5,10,11]. [^11^C]methyl iodide ([^11^C]CH_3_I) is used as the radioactive precursor in these radiotracer syntheses. [^11^C]choline ([^11^C]CHL) is used to evaluate suspected biochemically recurrent prostate cancer [12], whereas L-[^11^C]methionine ([^11^C]MET) has shown its potential in the clinic and research as an essential amino acid carrier for multiple metabolic pathways [13]. It has been used to determine staging, assess prognosis, and evaluate the response to therapy in multiple myeloma [14] and brain tumors [15].

The syntheses of [^11^C]MET and [^11^C]CHL using [^11^C]CH_3_I have been established [16,17,18,19]. However, conventional synthesizers are not cost-effective and require high precursor amounts [20,21,22]. Improvements in these requirements necessitate technological advancements to replace the existing synthesizers with upgraded models. This involves integrating state-of-the-art microfluidic techniques into radiosynthesizers to enhance their practicality in preclinical and clinical applications. Therefore, we explored a batch-type iMiDEV^TM^ microfluidic radiosynthesizer’s ability to produce different radiotracers and their adaptability to routine production using the same module [23,24,25]. The iMiDEV^TM^ module demonstrated that using 3 to 5 times less precursor allowed it to produce a comparable radiochemical yield to conventional modules [24,25,26,27,28].

In this study, we aimed to synthesize [^11^C]MET and [^11^C]CHL using a microfluidic cassette-based iMiDEV^TM^, a fully automated radiochemistry synthesis module, to assess compatibility in a single-dose production. We focused on optimizing the precursor amount for both radiotracers and explored their synthesis in microfluidic cassettes.

## 2. Results

The application of the microfluidic cassette-based iMiDEV™ module under optimized experimental conditions resulted in promising outcomes for synthesizing [^11^C]MET and [^11^C]CHL. After the optimization of the parameters, such as the quantities of precursor and radioactivity, the precursor loading conditions, and the microfluidic cassette, the fully automated syntheses were performed during the validation runs. The starting activity of [^11^C]CH_4_ was 23 GBq and 55 GBq for a 2 min and 5 min beam, respectively. [^11^C]CH_3_I was 5.5 GBq (5 min beam; 35µA) for both tracers. The precursor amount was optimized for [^11^C]MET to 400 µg. The radiochemical yield (RCY) was 84 ± 4%, and the synthesis time was 18 ± 1 min (*n* = 3). Similarly, the optimized precursor volume for [^11^C]choline was 25 µL, and the RCY was 66 ± 2%. The synthesis time was 24 ± 1 min (*n* = 3). The total product volumes of [^11^C]MET and [^11^C]CHL were 7.8 and 7.5 mL, respectively. The details of the RCYs of [^11^C]MET and [^11^C]CHL are summarized in Table 1.

The quality control (QC) results for [^11^C]MET and [^11^C]CHL demonstrated adherence to the acceptance criteria, ensuring the reliability and suitability of the synthesized radiotracers for clinical applications. High-performance liquid chromatography (HPLC) analysis confirmed that the radiochemical purities were >96.0% for [^11^C]MET and >99.0% for [^11^C]CHL. The HPLC chromatograms for both radiotracers are provided in Supplementary Materials, SM (Figures S1 and S2). The stability of the products was analyzed after 90 min, and the purity was >95% for [^11^C]MET and >99% for [^11^C]CHL. These comprehensive QC assessments highlight the precision and consistency achieved in synthesizing [^11^C]MET and [^11^C]CHL, thus reinforcing their viability for clinical use. A comprehensive overview of all the QC test results is presented in Table 2.

## 3. Discussion

**Figure 1:**
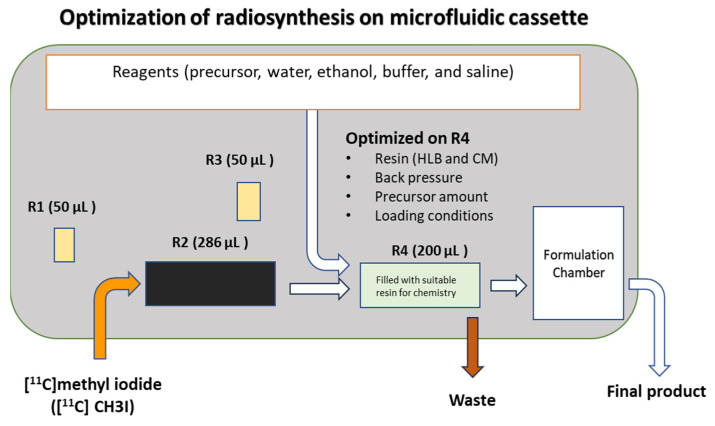
A summary of optimized conditions on R4 on the microfluidic cassette.

We are evaluating the implementation of the automated on-demand single-dose production of radiotracers tailored to clinic requirements. With a library of clinically relevant radiotracers, our objective is to supply these tracers to the clinic as needed to meet patient demands. In this study, we investigated the suitability of two of these tracers for on-demand single-dose production. The complete optimization of [^11^C]MET and [^11^C]CHL synthesis was performed using a microfluidic cassette. To do so, a new microfluidic cassette was used for each optimization run. Different types of resins (filled on R4) were tested for both radiotracers because of their unique chemistry. The microfluidic cassette with reagents was inserted on the iMiDEV^TM^ module, and the precursor was loaded on R4. Then, [^11^C]CH_3_I was trapped on R4, and the reaction was performed. Later, the product was extracted from R4 into a sterile product vial. The synthesis of [^11^C]MET and [^11^C]CHL involved the exploration of the microfluidic cassette, particularly reactor 4 (R4) (Figure 1), and optimization of the precursor amount (L-homocysteine thiolactone and dimethylaminoethanol), loading pressure, and time. The synthesis process is comprehensively automated and does not require any tube handling or specific know-how (including precursor loading, radiolabeling, SPE purification, or formulation). A summary of the optimized conditions for radiosynthesis ([^11^C]MET and [^11^C]CHL) on R4 in the microfluidic fluidic cassette is presented in Figure 1.

### 3.1. Microfluidic Cassette

The microfluidic cassette is one of the main factors influencing the optimization of the synthesis process. There are four reactors in the cassette; three of them (R1, R3, and R4) are used for on-column reactions at room temperature, while reactor 2 is used for reactions at elevated temperatures. In this study, only R4 was chosen to strategically minimize precursor contamination and decrease the overall synthesis time. R1 and R3 have a 50 µL capacity, while R4’s is 200 µL. Reactor 4 was investigated for both [^11^C]MET and [^11^C]CHL syntheses using HLB, C18, and CM resins. The back pressure at R4 and the precursor loading pressure and time were optimized to enhance the RCY. The back pressure on R4 is pivotal for precursor loading and thus for [^11^C]CH_3_I trapping. The bead density can significantly influence the reaction kinetics, thereby affecting the overall yield.

In the initial tests of [^11^C]MET synthesis, cassettes filled with C18 and HLB with back pressures between 800 and 870 mbar were chosen, and the precursor loading pressure was fixed at 200 mbar for 35 s. The radiochemical yield fluctuated between 50 and 86% (*n* = 6). The precursor loading on R4 with a fixed pressure and time was carefully monitored to observe the difference between the yields when using cassettes with back pressures of 800 and 870 mbar. Thereafter, tests were conducted by extending the precursor loading time to 50 s while maintaining a cassette back pressure of 853–877 mbar. However, this alteration did not yield stable results (RCY 39–72%; *n* = 5). Surprisingly, this adjustment did not improve the radiochemical yield; the RCY was lower than that of the previous method. These fluctuations and lower yields were due to the poor trapping of [^11^C]CH_3_I, and the poor precursor dispersion (high probability) on R4 could be explained by the high back pressure and also the increased time, which may have pushed the precursor out of R4 (although there is a negligible chance of this). After these tests, we determined that the density of the beads within them played a pivotal role in the synthesis process. A comparison of the yields also revealed substantial differences, highlighting the importance of cassette selection for synthesis optimization.

We selected only the HLB resin (60 µm) cassettes with similar back pressures (850–870 mbar) for further investigation. Furthermore, we optimized the pressure and time for precursor loading. This aspect is crucial because it directly affects the radiochemical yield. The precursor loading pressure and time were 200 mbar and 30 s, respectively. Under these conditions, the radiochemical yield was stable (RCY 83 ± 3%; *n* = 5).

For [^11^C]CHL, CM resin was used because of its suitable properties of high selectivity and sensitivity to strongly basic compounds. We applied the same precursor loading conditions, because ethanol served as the reaction solvent despite the R4 back pressure being approximately 850–900 mbar. However, these conditions did not yield the expected results, resulting in a RCY of 27 ± 15% (*n* = 3). The higher back pressure at R4, attributed to the CM resin (37–55 µm), can explain this outcome. Subsequently, by optimizing the pressure and time to 150 mbar and 15 s, respectively, and using a lower-volume vial (for the precursor: position F; 300 µL) instead of a 1.5 mL vial, we achieved a consistent and reproducible RCY of 60 ± 7%; *n* = 7.

The details of the optimized parameters for the syntheses of [^11^C]MET and [^11^C]CHL are summarized in Table 3.

### 3.2. Amount of Precursor and Reaction Time

Different precursor amounts and reaction times were tested to determine their influence on the radiochemical yield. For [^11^C]MET synthesis, 1.5 mg of L-homocysteine thiolactone hydrochloride in 500 µL of ethanol were used for the initial test, and the RCY_dc_ was 76% (*n* = 1) and the reaction time was 2 min. After this test, we focused on decreasing the amount of precursor used. We started with 400–500 µg of precursor, based on our previous experience with different tracers [24]. However, the volume of the precursor vial was maintained at 300 µL because of the larger reactor size (R4 is approximately 200 µL), and the reaction time was increased to 3 min. The radiochemical yield was 62 ± 17% (*n* = 3) when the precursor quantity was 400–500 µg. We also used 70% ethanol as the reaction solvent to improve the radiochemical yield; however, there was no increase in the radiochemical yield of 61 ± 8% (*n* = 3). When the precursor loading pressure and time were optimized, consistent RCYs were achieved (83 ± 3%; *n* = 5). There was no improvement in the yield when the reaction time was increased. The RCYs were between 79 and 88% when the precursor amount was optimized to 400 µg and the loading conditions were optimized (200 mbar, 30 s).

**Figure 2:**
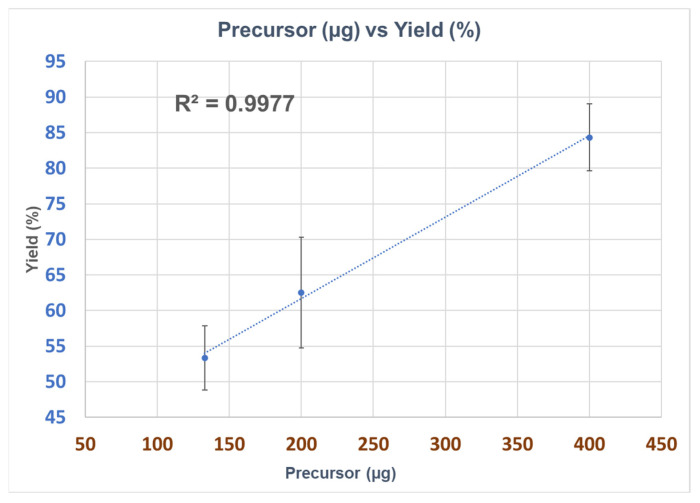
Visualization of radiochemical yield of [^11^C]*L*-methionine with respect to precursor amount, applying the optimized conditions for radiosynthesis. The precursor volume was 300 µL; the loading conditions were 200 mbar and 30 s; and the reaction time was 2 min.

A Further reduction in the precursor amount was performed to investigate the RCY, and the reaction time was maintained at 3 min. The details of the amounts of precursor used with their respective RCYs are summarized in Table 3. When the precursor amount was decreased from 400 µg to 200 µg, the yield gradually decreased from 83 ± 3% (*n* = 5) to 63 ± 6% (*n* = 2). When the precursor amount was further reduced to 133 µg, the RCY decreased to 54 ± 4% (*n* = 2). These results indicate that lowering the amount of precursor leads to a decrease in the precursor concentration for the reaction, reducing the overall RCY. An illustration of the decline in the RCYs corresponding to variations in the precursor quantity is shown in Figure 2. The yield decreased linearly when the precursor quantity was decreased from 400 µg to 133 µg. Notably, the precursor concentration was maintained by reducing the precursor volume, because a decreased precursor concentration correlated with lower RCYs. When 133 µg of precursor was used in 100 µL and 200 µL volumes, the RCYs were 54 ± 4% and 34 ± 15%, respectively. The main reason for the lowered yield was the reduction in the precursor’s concentration, resulting in a poor coating of the precursor on the resin within R4. It is important to maintain the homogeneity of the precursor in R4 to achieve consistent RCYs. Finally, the precursor amount was optimized to 400 µg with a 300 µL volume, and validation runs were performed using the same amount of precursor and the same volume, which was five times less than that of conventional radiosynthesizers [20,21,22,27,29]. The total synthesis time was 18 min.

For [^11^C]CHL, an initial trial was conducted using 100 µL (89 mg) of DMAE in 200 µL of ethanol with a reaction time of 2 min, yielding an RCY_dc_ of 49% (*n* = 1). Subsequent investigations aimed to determine the impact on the RCY by reducing the precursor volume from 100 µL to 12 µL of DMAE. An initial decrease in the yield was observed when the precursor volume was reduced to 50 µL. However, after optimizing the precursor loading conditions, the RCY increased to 60% (*n* = 4), surpassing the yield obtained when using 100 µL. RCYs of 58 ± 2% (*n* = 4) and 37±7% (*n* = 3) were obtained with precursor volumes of 25 µL and 12 µL, respectively, and the RCY decreased as the precursor volume decreased. This phenomenon was attributed to the lower precursor concentration in R4, which increased the unreacted [^11^C]CH_3_I and was observed during washing with ethanol and water. Notably, no significant difference in yield was observed between 25 and 50 µL. Consequently, the final precursor volume was optimized to 25 µL, which is 2–4 times less than the conventional method [20,27].

### 3.3. Amount of Radioactivity

The effect of the amount of radioactivity used in the synthesis on the radiochemical yield was also investigated. The complete details of the radiochemical yield obtained using different beam times with optimized synthesis conditions are summarized in Table 4. In the initial exploration of [^11^C]MET and [^11^C]CHL, a 2 min beam was employed, yielding an approximate [^11^C]CH_3_I activity of 2.5–3 GBq (*n* = 3). Subsequently, the optimization required an extended beam time of 5 min, which increased the [^11^C]CH_3_I activity (5.5 GBq; *n* = 3) to influence the RCY. Interestingly, there was no significant difference in RCY when transitioning from a 2 min to a 5 min beam; the difference was negligible. Specifically, using a 2 min beam for [^11^C]MET synthesis yielded 76 ± 7% (*n* = 3), while with a 5 min beam the yield slightly increased to 82 ± 3% (*n* = 4), indicating minimal variation between the two beam times. Similarly, for [^11^C]CHL, the comparison of RCY with beam time revealed a pattern similar to that of [^11^C]MET, where the 2 min beam yielded 58 ± 2% (*n* = 4) and the 5 min beam yielded 66 ± 2% (*n* = 3). An empirical comparison of the yields of [^11^C]MET and [^11^C]CHL indicated that the amount of radioactivity did not influence the overall RCY.

The optimized synthesis conditions, such as resin type, R4 back pressure, precursor amount, and loading conditions, and RCYs for both [^11^C]MET and [^11^C]CHL, are summarized in Table 5.

### 3.4. Comparison of iMiDEV Module with Conventional Module

The iMiDEV^TM^ batch-type microfluidic cassette-based synthesis module outperforms conventional synthesizers with its integrated design, reduced reagent volumes, precise control, and improved reproducibility. The iMiDEV^TM^ system offers an integrated platform with all essential synthesis components, including reactors, reagent reservoirs, and fluidic channels, seamlessly incorporated into a single disposable cassette. This design unlike conventional synthesizers, which typically entail bulkier components and manual intervention for cassette setup, necessitating more extensive setup and space requirements. Additionally, the iMiDEV^TM^ module employs significantly smaller volumes of reagents (100 µL) and precursors (4–5 times less than conventional modules), resulting in reduced consumption, cost-effectiveness, and minimized waste generation compared to conventional methods. The microfluidic nature of the iMiDEV^TM^ system allows for precise control over the reaction parameters, resulting in the improved reproducibility and reliability of tracer synthesis. While these significant advancements make iMiDEV^TM^ superior to conventional synthesis modules, it is important to acknowledge that while the iMiDEV^TM^ module represents a substantial advancement in microfluidic technology, it may not fulfill all the requirements demanded of the current radiotracers production. Nevertheless, ongoing advancements in microfluidic engineering, coupled with the modular and adaptable design of the iMiDEV^TM^ system, hold promise for overcoming current limitations and further enhancing the efficiency and versatility of radiotracer production, and are promising for future movement towards dose-on-demand production. Some of the other advantages of the iMiDEV^TM^ module over conventional modules are summarized in Table 6.

### 3.5. Study Limitations and Future Perspectives

This research contributes to the PET radiosynthesis methodology by optimizing the synthesis procedures for [^11^C]MET and [^11^C]CHL using a batch-type microfluidic iMiDEV™ synthesis setup. The synthesis model introduced in this study addresses the critical challenges in patient-centric radiotracer production and bolsters on-demand single dose production. While these accomplishments are noteworthy, it is essential to recognize that there are certain limitations, including the study’s focus on specific tracers. Future research should extend beyond [^11^C]MET and [^11^C]CHL, encompassing a broader spectrum of radiopharmaceuticals. This expansion aims to enhance the applicability of the dose-on-demand synthesis approach, while concurrently developing synthesis methods that minimize the need for extensive quality control tests. Ongoing efforts to refine microfluidic cassette parameters and explore novel resins will further contribute to the versatility and efficiency of this synthesis method. Commitment to continuous innovation and refinement remains pivotal for advancing PET imaging methodologies.

## 4. Materials and Methods

[^11^C]methane ([^11^C]CH_4_) was produced using a PET Trace 16.4 MeV Cyclotron from General Electric, Uppsala, Sweden. The microfluidic cassettes and the iMiDEV^TM^ radiosynthesizer were supplied by PMB-Alcen, France. Ethanol (99.5%) was procured from Kiilto Clean AB (Malmö, Sweden). Water (18 MOhm) was obtained using an in-house Milli-Q water purification system (Merck Millipore, Germany). Sterile water and sodium chloride (NaCl 0.9%) were purchased from B Braun, Melsungen, Germany. Sterile filters (Millex GV, 0.22 µm, 33 mm) and vent filters (Millex FG, 0.2 µm, 25 mm) were purchased from Merck Millipore (Carrigtwohill, Ireland). Dimethylaminoethanol (DMAE), L-methionine, and L-homocysteine thiolactone hydrochloride were purchased from Sigma-Aldrich (Darmstadt, Germany). Sodium hydroxide (NaOH), methanol (CH_3_OH), potassium dihydrogen phosphate (KH_2_PO_4_), sodium dihydrogen phosphate (NaH_2_PO_4_), and MQuant pH indicator strips were obtained from Merck KGaA (Darmstadt, Germany). Wheaton^®^ W986212NG NextGen™ V Vial^®^ 0.3 mL Clear Glass High Recovery Vials used in syntheses were supplied by Wheaton, USA. Additionally, 1.5 mL vials (V-shaped bottom), inserter vials (300 µL), and aluminum seals with septa (11 mm) were ordered from Thermo Scientific (Langerwehe, Germany). Glass vials (4 and 15 mL) were acquired from the Nordic Pack (Nykvarn, Sweden). Sterile vacuum vials (15 mL) were procured from Huyai Isotopes Co. (Suzhou, China).

### 4.1. Radiosynthesis of L-[^11^C]Methionine and [^11^C]Choline

#### 4.1.1. Preparation of [^11^C]Methyl Iodide ([^11^C]CH_3_I)

[^11^C]CH_4_ was produced from a methane target via the ^14^N(p, α)^11^C nuclear reaction in a cyclotron. The target was filled with nitrogen gas mixed with 10% hydrogen and bombarded for 2–5 min at 35 µA. [^11^C]CH_3_I was synthesized using the TracerMaker module with in-target-produced [^11^C]CH_4_ as previously reported [30]. After the production of [^11^C]CH_3_I (~3–5.5 GBq), it was transferred through a separate line directly connected to the iMiDEV™ radiosynthesizer. The flow rate of [^11^C]CH_3_I was set at 8 mL/min [24].

#### 4.1.2. Automated Radiosynthesis

**Figure 3:**
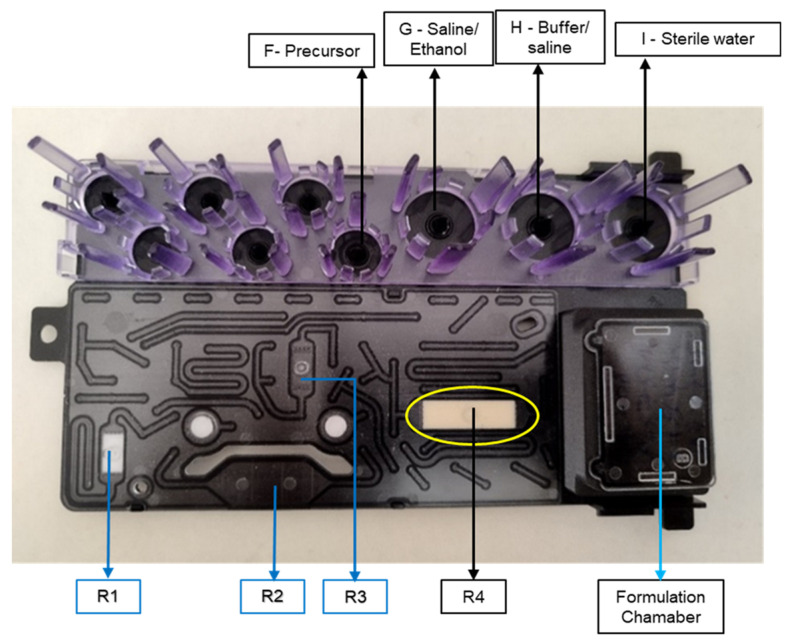
A microfluidic cassette for on-demand synthesis of L-[^11^C]methionine and [^11^C]choline.

iMiDEV™ is a batch-type microfluidic cassette-based radiosynthesizer that produces radiotracers at room and/or elevated temperatures [23,24,25]. This study used a single-use microfluidic cassette with suitable resins for different radiotracers. A picture of the microfluidic cassette with the vial position utilized in this study is shown in Figure 3. The entire synthesis process, including radiolabeling, solid-phase extraction (SPE) purification, and formulation, was integrated within the microfluidic cassette. All the synthesis steps were performed in auto mode without any manual intervention. A complete overview of the iMiDEV^TM^ supervision software is provided in the Supplementary Material (Figure S3). The iMiDEV™ radiosynthesizer is part of the iMiGiNE™ automated radiopharmaceutical production system.

All reagents used for the [^11^C]MET and [^11^C]CHL syntheses and their respective vial positions are summarized in Table 7, and the reaction schematics are provided in the Supplementary Material (Schemes S1 and S2). The procedure for back pressure measurement (flow resistance measurement) in the reactors (R1, R3, and R4) and the bead filling of the reactors was described in our previous publication [24]. The RCY was calculated by dividing the starting activity by the final obtained product activity. All the yields are decay-corrected unless otherwise mentioned.

#### 4.1.3. [^11^C]Methionine

The production of [^11^C]MET involved using the R4 chamber in the microfluidic cassette for the reaction and purification. The HLB (hydrophilic-lipophilic) beads facilitated the trapping of radioactivity in the R4 chamber, and the reaction was performed. The final product was eluted and diluted in the formulation chamber before being transferred to a sterile vial via a sterile filter and through an extraction valve.

**Figure 4:**
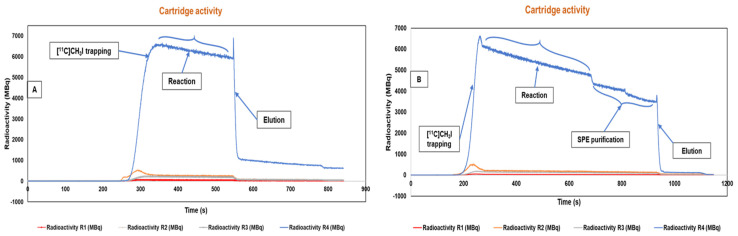
The radiosensor data of the automated complete (**A**) [^11^C]MET and (**B**) [^11^C]CHL radiosynthesis. Beam time was 5 min (~5.5 GBq of [^11^C]CH_3_I) for the both tracers.

For [^11^C]MET synthesis, L-homocysteine thiolactone (precursor) was mixed with 99.5% ethanol and 5 M sodium hydroxide. The mixture was vortexed for 3 min before loading into a microfluidic cassette at position F. At position H, 2 mL of 50 mM NaH_2_PO_4_ phosphate buffer and 6 mL of 0.9% NaCl saline was placed at position G. After all the reagents were loaded, the cassette was placed in the synthesis box, clamped, and pressurized prior to starting the synthesis. Synthesis was initiated by transferring the precursor from vial F to R4 by opening microfluidic valves (MFVs) 27 and 28 (Figure S3; SM) at a pressure of 200 mbar for 30 s. Once [^11^C]CH_3_I reached the detectors of R2 from the TracerMaker module through opened MFVs 8, 12, 13, 18, and 20 (Figure S3; SM), radioactivity was channeled towards the R4 chamber by closing MFV 20 and opening MFVs 22 and 28 (Figure S3; SM). After the maximum radioactivity was trapped in R4 (Figure 4), MFV 28 was closed, and the reaction proceeded for 3 min. Following the reaction, the product was eluted with phosphate buffer (NaH_2_PO_4_) from vial H into the formulation chamber for further dilution. MFVs 24, 25, 29, 33, and 34 (Figure S3; SM) were opened at a pressure of 1500 mbar for 90 s. A similar process was repeated with vial G through MFVs 26, 29, 33, and 34 (Figure S3; SM) for dilution of the product with 0.9% NaCl saline at a pressure of 1500 mbar for 60 s.

After all these synthesis steps, including the radiochemical reaction, SPE purification, and formulation, the final product was transferred to a sterile 15 mL product vial through a 0.22 µm sterile filter. The radiosensor data of the complete synthesis of [^11^C]MET and [^11^C]CHL are provided in Figure 4.

#### 4.1.4. [^11^C]Choline

The synthesis was initiated by transferring the precursor from vial F to R4 (filled with CM resin), which was accomplished by opening the valves MFVs 27, 28 (Figure S3; SM) under a pressure of 150 mbar for 15 s. Once [^11^C]CH_3_I reached the detector of R2 from the TracerMaker module via the opened MFVs 8, 12, 13, 18, and 20 (Figure S3; SM), radioactivity was directed toward the R4 chamber valve by closing MFV 20 and opening MFV 22 and MFV 28. After trapping the maximum radioactivity in R4, MFV 28 was closed, and the reaction proceeded for 5 min. Following the reaction, R4 was washed with 3 mL of ethanol and sterile water and then eluted with 8 mL saline into the formulation chamber. MFVs 24, 25, 29, 33, and 34 (Figure S3; SM) were opened under a pressure of 1950 mbar for 120 s. After the synthesis steps were completed, including the radiochemical reaction, solid-phase extraction (SPE) purification, and formulation, the final product was transferred to a sterile 15 mL product vial through a 0.22 µm sterile filter.

The details of the whole synthesis preparation for [^11^C]MET and [^11^C]CHL, from the start to the end of the synthesis, are summarized in Table 8.

## 5. Conclusions

In conclusion, this study successfully optimized the synthesis of [^11^C]MET and [^11^C]CHL using a microfluidic cassette-based iMiDEV™ module, demonstrating its robustness for on-demand single-dose synthesis, which could fulfill a single patient’s requirements or be divided for multiple doses. The meticulous exploration of the microfluidic cassette parameters, including reactor 4, the resin types, and back pressure optimization, demonstrated their pivotal role in ensuring stable and reproducible RCYs. The optimized precursor amount for both [^11^C]MET and [^11^C]CHL was 4–5 times less than that of conventional radiosynthesizers, and the obtained final products were sufficient for a single patient dose from a lower starting activity. The impact of the amount of radioactivity on the synthesis revealed that an extended beam time did not significantly alter the yields of either tracer, thereby emphasizing the consistent performance of this synthesis approach utilizing a lower starting activity. Our synthesis process was fully automated, with the iMiDEV™ module seamlessly handling all of the synthesis steps under optimized reaction conditions. This significant step toward complete automation underlines the efficiency and reproducibility of our microfluidic cassette-based synthesis method, offering a promising pathway for a single-patient or multiple-dose production of these critical radiotracers for clinical applications. This systematic exploration and fine-tuning of the microfluidic synthesis process, along with the automated synthesis, offer a technical solution to advance toward fulfilling real-time, patient-specific radiopharmaceutical production. The study yielded [^11^C]MET of 3233 ± 154 MBq and [^11^C]CHL of 2368 ± 103 MBq from ~5.5 GBq of [^11^C]CH_3_I, confirming the efficacy of the optimized synthesis for clinical applications. Moreover, evaluating and implementing microfluidic modules in routine clinical and preclinical production to produce several other radiotracers is another path open for future research.

## Figures and Tables

**Table 1:** Summary of L-[^11^C]methionine and [^11^C]choline production from validation runs.

Radiotracer	Number of Production (*n*)	Product Activity (MBq)	Radiochemical Yield *	Radiochemical Yield ** (%)	Radiochemical Purity (%)
L-[^11^C]methionine	3	3233 ± 154	45 ± 2	84 ± 4	>96.0
[^11^C]choline	3	2368 ± 103	42 ± 2	66 ± 2	>99.0

* EOS; ** SOS; yields are determined by radio-HPLC analysis of the final product.

**Table 2:** Summary of quality control tests.

Parameters	Acceptance Criteria	[^11^C]*L*-Methionine	[^11^C]Choline
Appearance	Clear, colourless, free from particulates	Complies	Complies
pH	5–8.5	6.0	6.0
Filter integrity test	>3.5 bar	4.0	3.8
Radiochemical purity	>95% by HPLC	>96.5	>99.0
Radionuclide identity	T_½_ = 19.9–20.9 min	20.4 ± 0.20	20.2 ± 0.10
Ethanol (GC)	<10%	<2.0	<2.0
Bacterial endotoxins (EU/mL)	<17.5	<5.0	<5.0
L-homocysteine	<200 µg/mL	11 ± 2	NA
Dimethylaminoethanol	<1000 µg/dose *	NA	155 ± 50

EU—endotoxin units; GC—gas chromatography; * <133 µg/mL; NA—not applicable.

**Table 3:** Summary of optimized conditions like resin type and size, back pressure, precursor amount, and obtained RCY.

Radiotracers	Resin Type	Resin Size (µm)	R4 Back Pressure (mbar)	Loading Conditions	Precursor Amount	RCY (%)
L-[^11^C]methionine	C18	55,105	803–810	200 mbar, 35 s	400 µg	51 ± 1% (*n* = 2)
	HLB	60	806–866	200 mbar, 35 s	400 µg	67 ± 13% (*n* = 4)
	HLB	60	853–870	200 mbar, 50 s	400 µg	66 ± 9% (*n* = 3)
	HLB	60	850–870	200 mbar, 30 s	400 µg	83 ± 3% (*n* = 5)
	HLB	60	850–870	200 mbar, 30 s	200 µg	63 ± 6% (*n* = 2)
	HLB	60	850–870	200 mbar, 30 s	133 µg	34 ± 15% (*n* = 2)
[^11^C]Choline	CM	37–55	901	200 mbar, 30 s	100 µL ^a^	49% (*n* = 1)
	CM	37–55	858–905	200 mbar, 30 s	100 µL	27 ± 16% (*n* = 3)
	CM	37–55	886–909	150 mbar, 30 s	50 µL	60 ± 4% (*n* = 4)
	CM	37–55	881–907	150 mbar, 15 s	25 µL	58 ± 2% (*n* = 4)
	CM	37–55	896–915	150 mbar, 15 s	12 µL	37 ± 7% (*n* = 3)

Precursor volume was 300 µL for the all L-[^11^C]methionine syntheses reported in this table. For [^11^C]Choline, ^a^: precursor volume was 300 µL, while the rest of the syntheses’ precursor volumes were 200 µL. Ethanol was used as the reaction solvent for the tracers.

**Table 4:** Summary of radiochemical yield using different beam time with optimized synthesis conditions.

Radiotracers	No of Syn (*n*)	Beam Time (min)	Product Activity (MBq)	Radio Chemical Yield (%)
L-[^11^C]methionine	3	2	1681 ± 340	76 ± 7
	4	5	3343 ± 281	82 ± 3
[^11^C]choline	4	2	938 ± 37	58 ± 2
	3	5	2368 ± 103	66 ± 2

**Table 5:** Summary of optimized conditions for [^11^C]MET and [^11^C]CHL synthesis.

Tracers	Resin Type	R4 Back Pressure (mbar)	Precursor Amount	Loading Conditions	RCY (%)
L-[^11^C]methionine	HLB	850–870	400 µg	200 mbar, 30 s	83 ± 3% (*n* = 5)
[^11^C]Choline	CM	890–905	25 µL	150 mbar, 15 s	60 ± 7% (*n* = 7)

**Table 6:** Comparison of various aspects of iMiDEV^TM^ and conventional synthesis modules.

Aspects	iMiDEV^TM^ Microfluidic Module	Conventional Modules
Cassette	Microfluidic cassette-based system with tubing-free design	Traditional cassette-based system with manual connections with tubing
Reagent Transfer	Allows for precise control of reagent flows	Reagents are added either automatically or manually
Precursor	Requires 4–5 times less precursor compared to conventional modules	Often necessitates more precursor (1–5 mg)
Radioactivity	Low starting activity	Normally requires high starting activity
Radiation Safety	Enhanced safety due to lower activity levels	Adheres to standard safety protocols or increases radiation exposure for production chemist
Synthesis Automation	Offers fully automated synthesis process	Synthesis process can be automated/semi-automated/manual
Continuous Improvement	Allows for ongoing optimization and updates	Offers limited scope for continuous enhancements
DOD or Single Dose Production Support	Facilitates single dose production with minimal intervention	Feasible, but expensive for single dose production
Production Cost	Lower due to reduced consumption of reagents	Higher due to the increased consumption of reagents

**Table 7:** List of reagents used for L-[^11^C]methionine and [^11^C]choline synthesis.

Vial Position	L-[^11^C]Methionine	[^11^C]Choline
Vial F	400 µg of precursor in 300 µL ethanol	22 µL of precursor in 125 µL ethanol
Vial G	6 mL saline	3 mL ethanol
Vial H	2 mL phosphate buffer	8 mL saline
Vial I	N/A	3 mL sterile water

**Table 8:** Summary of the optimized synthesis steps for L-[^11^C]methionine and [^11^C]choline.

Steps	Duration	Description
**Pre-synthesis**	30 min before EOB	Preparation of the microfluidic cassette and reagents for the synthesis.
**Precursor loading on reactor 4 (R4)**	For [^11^C]MET 200 mbar, 30 s For [^11^C]CHL 150 mbar, 15 s	Vial F used for both tracers. Precursor volumes of 300 µL (400 µg of prec) and 150 µL (25 µL of prec) were used for [^11^C]MET and [^11^C]CHL, respectively.
**Radiolabeling**	EOB + 10–14 min	The [^11^C]CH_3_I line was connected to iMiDEV from the TracerMaker module and transferred to iMiDEV using 8mL/min of helium gas. When the maximum activity was trapped on R4, the reaction was started.
**Reaction time**	For [^11^C]MET—3 min For [^11^C]CHL—5 min	By closing all the valves, the reaction was started at room temperature.
**SPE purification**	4–5 min ^a^	For [^11^C]CHL, to remove unreacted precursor and [^11^C]CH_3_I, R4 was washed with 3 mL ethanol followed by 3 mL sterile water. [^11^C]MET: No SPE purification.
**Product elution and formulation**	4 min	For [^11^C]MET, phosphate buffer used to elute, followed by formulation with saline. For [^11^C]CHL, only saline was used. Finally, the product was filtered through a 0.22 µm sterile filter.

^a^ Only for [^11^C]CHL; there was no SPE purification for [^11^C]MET.

## Data Availability

The datasets used and/or analyzed during the current study are available from the corresponding author upon reasonable request.

## Supplementary Materials

The following supporting information can be downloaded at https://www.mdpi.com/article/10.3390/ph17020250/s1, Figure S1: HPLC chromatogram of L-[^11^C]methionine (radio and UV detector); Figure S2: HPLC chromatogram of [^11^C]choline (radio and refractive index detector); Scheme S1: schematic of L-[^11^C]methionine reaction; Scheme S2: schematic of [^11^C]choline reaction; Figure S3: an overview of the iMiDEV^TM^ supervision software (Version supervision 1.0.2.12).
