# Supplementary material for: Advancements in Microfluidic Cassette-Based iMiDEV™ Technology for Production of L-[11C]Methionine and [11C]Choline
Source: Pharmaceuticals (Basel). 2024 Feb 15;17(2):250. doi: 10.3390/ph17020250 (PMC10891588; doi:10.3390/ph17020250)
Supplement: Supplementary file 1 [file pharmaceuticals-17-00250-s001.zip › pharmaceuticals-2842834-supplementary.pdf]

Supplementary Information

Advancements in microfluidic cassette-based iMiDEV™ technology for production of L-[^{11}C]Methionine and [^{11}C]Choline

Hemantha Mallapura [1], Laurent Tanguy [2], Samin Mahfuz [1] , Lovisa Bylund [3], Bengt Långström [4], Christer Halldin [1] and Sangram Nag [1,*]

[1] Department of Clinical Neuroscience, Center for Psychiatry Research, Karolinska Institutet and Stockholm County Council, SE-17176, Stockholm Sweden; hemantha.mallapura@ki.se (H.M.); christer.halldin@ki.se (C.H.); sangram.nag@ki.se (S.N.); mahfuz@kth.se (S.M)

[2] Business Unit Nuclear Medicine, PMB-Alcen, Route des Michels CD56, F-13790 Peynier, France; ltanguy@pmb-alcen.com (L.T.)

[3] Department of Radiopharmacy, Karolinska University Hospital, SE-17176, Stockholm Sweden; lovisa.bylund@regionstockholm.se (L.B.)

[4] Department of Medicinal Chemistry, Uppsala University, Uppsala 75123, Sweden; bengt.langstrom@kemi.uu.se (B.L.)

* Correspondence: sangram.nag@ki.se

Corresponding author:

Sangram Nag

Department of Clinical Neuroscience

Center for Psychiatry Research

Karolinska Institutet and Stockholm County Council

SE-171 76, Stockholm, Sweden.

E-mail: sangram.nag@ki.se

Content

Figure S1. HPLC chromatogram of L-[^{11}C]methionine (radio and UV detector). – Page 3

Figure S2. HPLC chromatogram of [^{11}C]choline (radio and refractive index detector).- Page 3

Scheme S1. Schematic of L-[^{11}C]methionine reaction – Page 4

Scheme S2. Schematic of [^{11}C]choline reaction – Page 4

Figure S3. An overview of the iMiDEV™ supervision software- Page 5

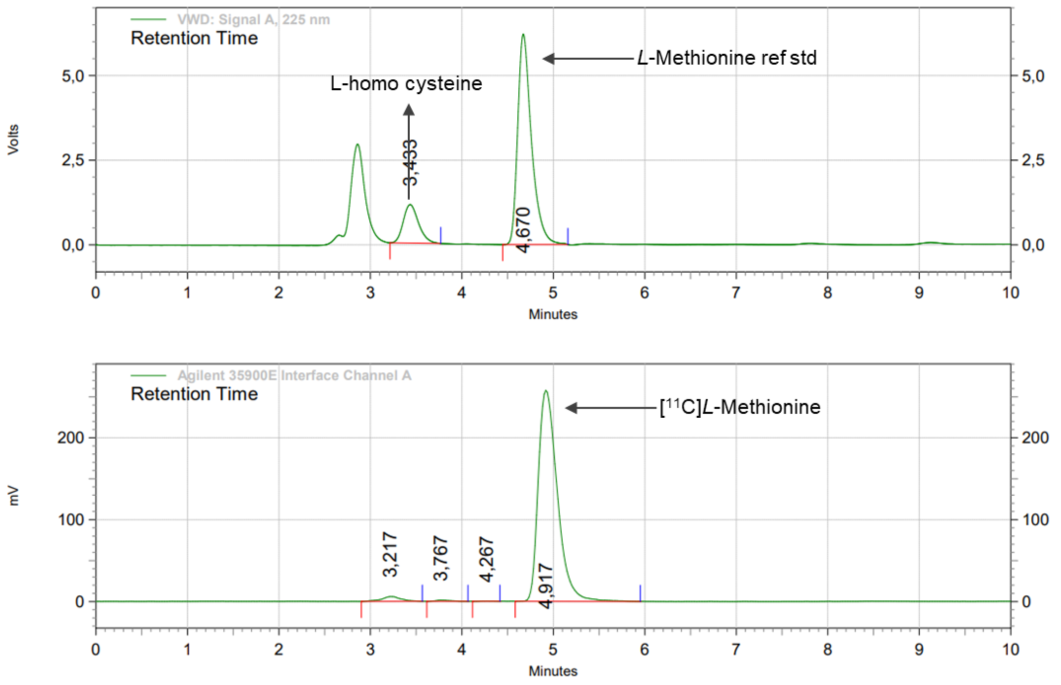

Figure S1. HPLC chromatogram of L-[11C]methionine (radio and UV detector).

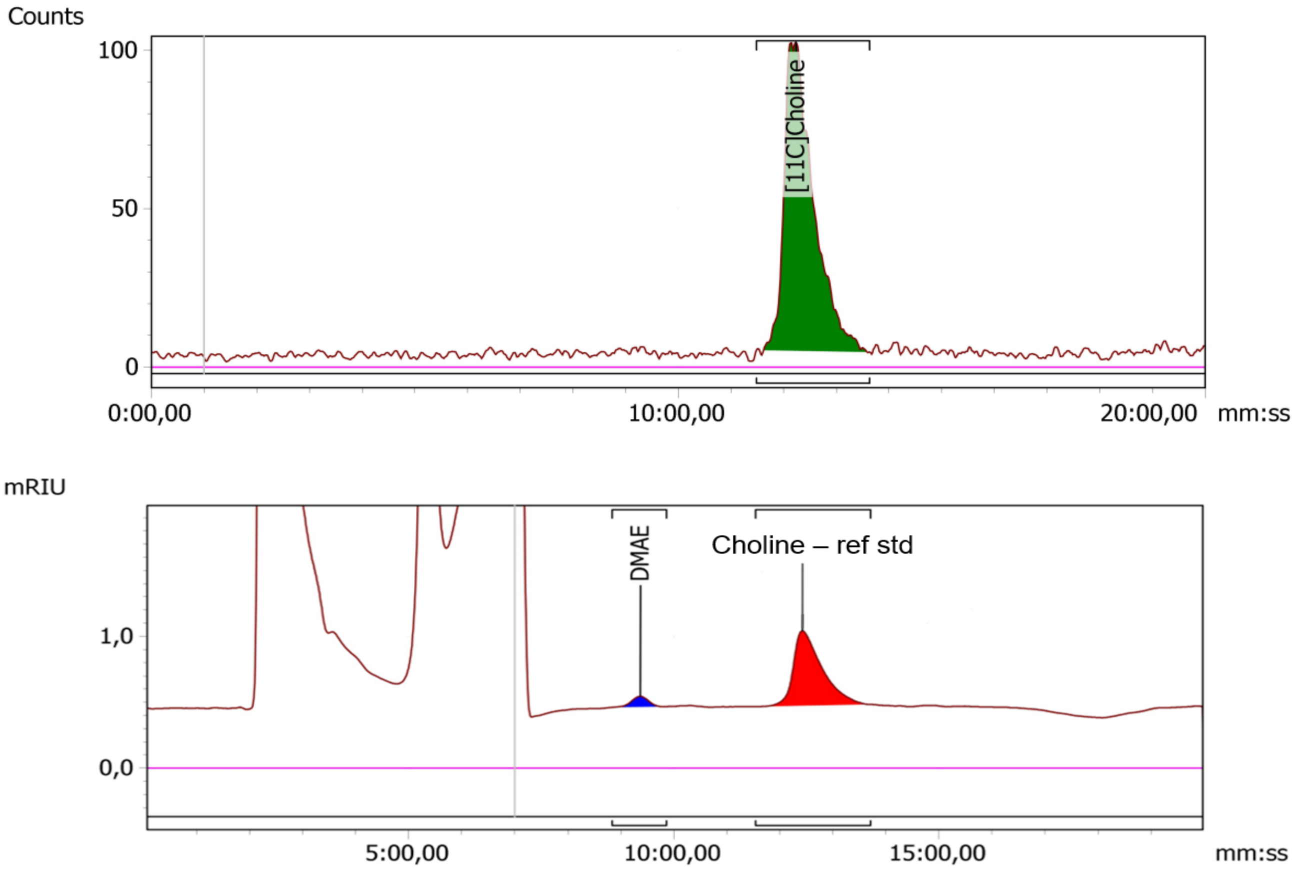

Figure S2. HPLC chromatogram of [11C]choline (radio and refractive index detector).

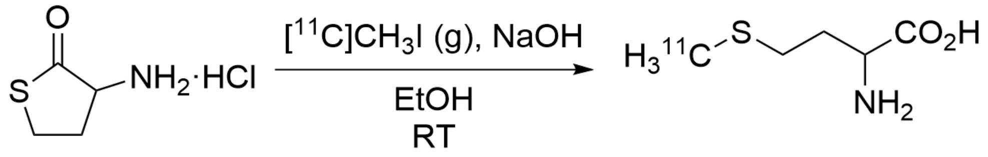

L-homocysteine thiolactone

L-[11C]methionine

Scheme S1. Schematic of L-[11C]methionine reaction

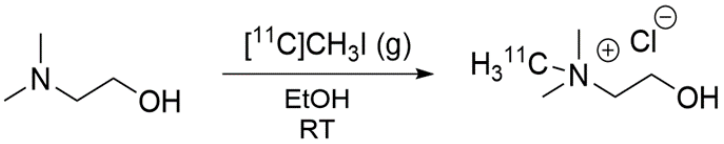

2-Dimethylaminoethanol

[11C]Choline

Scheme S2. Schematic of [11C]choline reaction

Figure S3. An overview of the iMiDEV™ supervision software

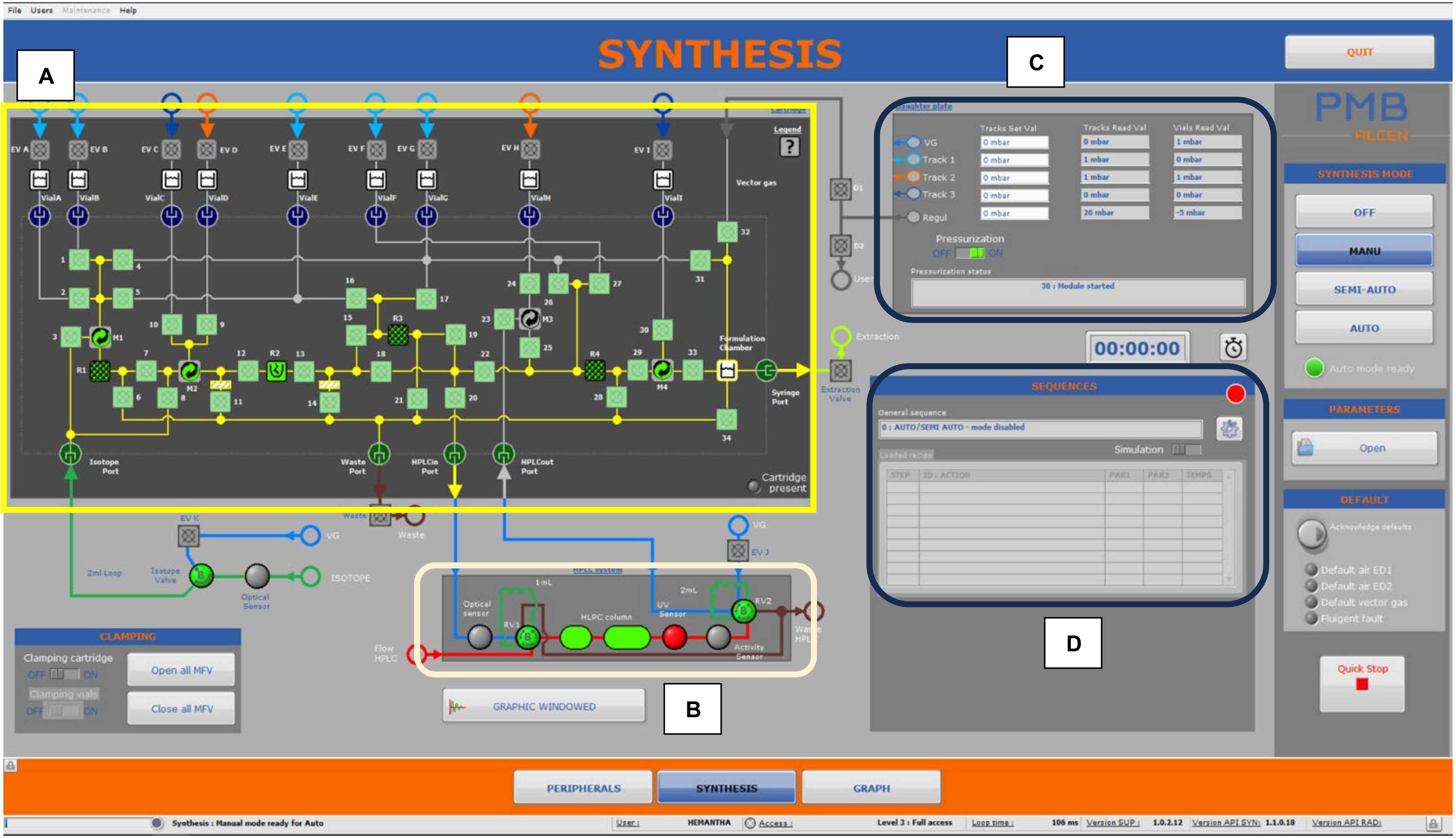

The above figure shows the complete configuration of the iMiDEV™ microfluidic module including, A) 34 microfluidic valves, B) preparative HPLC connected to the cassette for purification, C) pressure regulation reagents transfer , and D) sequence loading tab.
